# Variation in patient-sharing network characteristics of health care professionals treating different mental and substance use disorder patient sub-groups in primary care

**DOI:** 10.1177/00207640241270827

**Published:** 2024-08-30

**Authors:** Marko Elovainio, Laura Hietapakka, Mai Gutvilig, Ripsa Niemi, Kaisla Komulainen, Laura Pulkki-Råback, Visa Väisänen, Timo Sinervo, Christian Hakulinen

**Affiliations:** 1 Department of Psychology and Logopedics, Faculty of Medicine, University of Helsinki, Finland; 2 Finnish Institute for Health and Welfare, Helsinki, Finland

**Keywords:** Social networks, patient-sharing, primary care, psychiatry, register study

## Abstract

**Background:**

Providing efficient and targeted services for patients with mental health problems requires efficient collaboration and coordination within healthcare providers, but measuring collaboration using traditional methods is challenging.

**Aims:**

To explore the patient-sharing networks of professionals taking care of different groups of patients with mental or substance use disorders.

**Method:**

We used data that covered adult patients’ visits to the primary care service providers of seven municipalities in Finland during year 2021. Data included 8,217 patients (147,430 visits) with mental or substance use disorders who were treated by 1,566 health care professionals. We calculated descriptive network metrics to examine the connectivity of professionals in three different patient groups (patients with substance use disorders, psychotic disorders, and depressive disorders) and compared these characteristics to a network based on all patients. We also analyzed whether patient sharing was associated with the health care professionals’ attributes (occupational group, municipality) using Exponential Random Graph Models (ERGM).

**Results:**

Diagnosis-specific networks were denser and more connected compared to the all-patients network. Nurses were the most central occupation in all the diagnosis-specific networks and especially in the substance use disorder patients network. When examining all patients, two professionals were more likely to share patients when they belonged to the same occupational group. However, in the network with depressive disorder patients we found the opposite: professionals were more likely to share patients if they were of different occupational groups.

**Conclusions:**

Patient-sharing networks within patients with a specific mental or substance use disorders are denser and more connected than networks based on all patients with mental or substance use disorders. In the substance use disorder patients network particularly, nurses were the most central occupation. Multi-professional connections were more likely in depressive disorder networks than in the all-patients network.

## Introduction

People with mental and substance use disorders are more likely to have comorbid mental disorders and somatic illnesses (Berk et al., 2023; Momen et al., 2020; Plana-Ripoll et al., 2019) and they often need higher levels of support in comparison to the general population (Firth et al., 2019). It is also relatively common for a mental disorder and substance use disorder to occur simultaneously (Kessler et al., 1996; Kuussaari & Hirschovits-Gerz, 2016; Melartin et al., 2002). Mental disorders have been shown to reduce the ability to access available health services and advocate for one’s own interests (Wix & Spigt, 2022) and people with substance use disorders often need particular attention in order to ameliorate accumulating social problems, which are common among them (Saunders et al., 2016).

To successfully treat people with mental and substance use disorders, a diverse range of health services delivered by various specialized health care professionals is required (Aylott et al., 2019; Olfson, 2016). Consequently, knowledge distributed across professionals, units, and organizations needs to be integrated and coordinated efficiently (Firth et al., 2019; Wahlbeck et al., 2018). Actions implemented in the service system must also encompass wider collaborative networks from a broader perspective including preventative actions, treatment, and rehabilitation. International trends in mental health service development lean toward more collaborative approaches involving more person-centered approaches and providing multiple services according to individual needs (World Health Organization [WHO], 2014).

Providing such services requires efficient collaboration and coordination between primary healthcare providers and specialized services. It is self-evident that patients with mental and substance use disorders require tailored treatment and care aligned with their specific needs and symptoms as acknowledged in clinical guidelines and recommendations (Addington et al., 2017; Moriana et al., 2017; Taylor & Cleare, 2021). It is reasonable to assume that sufficient collaboration and coordination should also be modified based on needs of patients and patient groups (WHO, 2021). However, the extent to which such adaptations are implemented remains unclear. According to the national strategy evaluation in Finland, for instance, the health services of those with mental health and substance use problems are fragmented, and service providers have failed to harmonize their activities, collaboration, and division of duties. This has then reduced the availability and allocation of services (Ministry of Social Affairs and Health, 2020).

Establishing effective collaboration in mental health care requires methods that can be used to identify and analyze the underlying structures and factors of health care professionals’ interactions. Traditionally, health care networks have been examined using surveys. More recently, social network analysis (SNA) has been used to explain cooperation and information exchange patterns among professionals, such as the diffusion of medical innovations (Coleman et al.,1966; Creswick & Westbrook, 2010; Wensing et al., 2010), decision making (Creswick & Westbrook, 2015; Scott et al., 2005), and organizational hierarchies (West et al., 1999). Previous studies conducted in mental health care have suggested for example that better continuity of care is associated with large, centralized, and homophilous networks (Lorant et al., 2017). Despite the extensive application of SNA in healthcare, comparatively less attention has been directed toward exploring patient related factors that may influence the formation of social networks among health care professionals in mental health care (Chambers et al., 2012). Previous studies have explored interorganizational or provider level networks in child or youth mental health (Bustos, 2020), inter-professional networks (Barnett et al., 2015; Pomare et al., 2019) or on specific age groups with mental illnesses (Breslau et al., 2021).

Several concerns remain about the mechanisms through which these networks emerge within healthcare organizations and whether and to what extent the network characteristics of health care professionals differ between patient groups. Using administrative data in exploring patient-sharing networks (i.e. networks that are based on patients that different professionals have in common) offers a possibility to analyze the connections between health care professionals without the common pitfalls of surveys and allows to examine how relationships between professionals are affected by patient characteristics (Saint-Pierre et al., 2020). However, most previous studies exploring patient-sharing networks using administrative data have focused on physician relationships and often in a hospital context without a focus on mental health services (DuGoff et al., 2018).

In the present study, we examined the relationships between all professionals taking care of patients with mental and substance use disorders in one area in the Finnish primary care during the year 2021. We considered all visits to primary care and additionally focused on three different patient groups (patients with substance use, psychotic, and depressive disorders) to explore the possible differences in patient sharing networks between the patient groups. In Finland, primary care mental health and substance abuse services are mainly offered by GPs, nurses, and mental health professionals or teams working in health and social centers or in occupational health care. Other services comparable to primary care (also included in our study) are provided by student health care services. From the other factors potentially affecting the professional connections in health care alongside the patient’s diagnosis, we considered health care professionals organization and occupation; professionals working in the same organization (run by the same municipality) or in the same occupation are more likely to interact reciprocally, because they have similar problems they need to share and solve (Mascia et al., 2015) for an exception, see Breslau et al., 2021).

## Methods

### Data and study population

The data were from the Register of Primary Health Care visits (Avohilmo) maintained by the Finnish Institute for Health and Welfare (THL), which cover all primary health care visits in Finland. We used a subsample that comprised visits to the primary care service providers of seven municipalities. We only considered visits that occurred between January 1st 2021 and December 31st 2021 among the patients aged 18 years or older who had at least one visit within one of the municipalities.

In the register each service user has a unique pseudonymized personal identification code. Using this code, we selected only those patients who had visited primary care services for reasons relating to mental health or substance abuse between January 1st 2021 and December 31st 2021. A visit was identified as being related to mental health or substance abuse if the information regarding the visit included (1) a psychiatric diagnosis based on the International Classification of Diseases, 10th revision (ICD-10) subchapter F (excluding dementia, mental retardation, and specific developmental disorders), (2) a psychological symptom or diagnosis based on the International Classification of Primary Care second edition (ICPC-2), subchapter P (excluding dementia, mental retardation, and specific learning problems), and/or (3) the visit’s service type was marked as related to mental health or substance abuse.

Patients with mental health or substance abuse related needs also often have other needs related for example physical health issues which are also addressed in primary care services. Thus, we included all the visits made by the chosen patient population to healthcare or social services in primary care. We excluded, however, visits that were delivered to the patients (home care visits), since these visits are mostly provided for elderly people needing help with daily practices or healthcare services at home. We also removed the visits if the service type was undefined. Visits to occupational health care were also excluded, because of lacking information on the health care providers’ attributes. Furthermore, we excluded visits to professionals who are unlikely to be involved in direct patient care such as secretaries, managers, pharmacists, and opticians.

Finally, we further excluded those patients, who only had one visit during the 1-year time period, as well as those patients, whose visits were all to the same (one) professional. This was done using each professional’s unique registration number. These exclusions were made as they would not add information on patient sharing. From the register we included information on municipality, occupational group of the professionals, and the service type. The initial data from year 2021 included 178,035 patients and 2,952,381 visits. After identifying and choosing only the patients that had had mental health or substance abuse related diagnose or visits, the data included 9,039 patients. After removing the home care visits and ‘the other’ visits the data included 162,122 visits. Removing visits with missing information on occupational identification number, occupation, or type of visit resulted in 159,375 visits. After removing visits to secretaries, managers, pharmacists, and opticians and after removing the patients who had only one visit or all their visits were to the same professional, the final data consisted of 8,217 patients and 147,430 visits to 1,559 different healthcare professionals. The Finnish Institute for Health and Welfare ethics committee approved the study (THL/5787/6.02.01/2021/§889), and the Finnish Institute of Health and Welfare permitted the data linkages.

### Patient sub-groups, visit, and health care professional characteristics

Using the overall data, that is patients with any mental disorder diagnosis or mental health care visits, we further extracted three different patient sub-group data (1) those with substance use disorders (ICD-10 diagnoses F10–F19), (2) those with psychotic disorders (ICD-10 diagnoses F20–F29), and (3) those with depressive disorders (ICD-10 diagnoses F30–F39). Similarly, as in the overall data, we included all visits these patients had, not only the ones related to this diagnosis.

We classified the service type of the visit into the following four categories: appointments related to (A), general care, (B) substance use or mental health care, and (C) to routine care (such as vaccinations) and (D) other visits.

The occupation of the health care professionals was classified as physicians, nurses (including registered nurses, practical nurses, and other nurses such as radiographers or community health nurses), and other professionals (including psychologists, physio- and other therapists, and social workers). If one professional had more than one registered occupation, the most frequently registered occupation was chosen. If the frequency of registered occupations was equal, one was randomly selected.

The municipality of the professional was defined as the municipality in which most of the visits were recorded. If a professional worked equally much in several municipalities, one was randomly selected.

### Statistical analyses

For each of the four patient groups, the following procedures were applied to construct the patient-sharing networks. From here on out, the network based on all mental health and substance use patients will be referred to as the all-patients network, and the specific diagnosis group networks will be referred to as the substance use, psychosis, and depressive networks.

The initial healthcare professional-patient networks consisted of connections between patients and the healthcare professionals they visited. Although included patients needed to have visited primary care services for reasons relating to mental health or substance abuse, all of their visits and all of the health professionals in each area were included. These kinds of networks are called bipartite networks, where there are two distinct sets of nodes (in this case healthcare professionals and patients) and ties can be formed only between the two sets, not within (Pavlopoulos et al., 2018). In these kinds of networks patients can only be connected to professionals, not other patients, and vice versa. To form unipartite patient-sharing networks, healthcare professionals were connected if they were both tied to at least one same patient. The resulting patient-sharing networks contain nodes (professionals) connected by edges (shared patients). Previous studies suggest that sharing only one or few patients does not necessarily imply co-operation. However, it has been shown that health care professionals who shared more patients were also more likely to report having a professional relationship (23). Thus, we considered two professionals to be connected if they shared at least five patients (DuGoff et al., 2018).

Networks were depicted using the Fruchterman-Reingold layout algorithm (Fruchterman & Reingold, 1991) with node color based on the occupation of the professional and node size reflecting degree centrality. The Fruchterman-Reingold algorithm may produce warped figures if the network contains a few disconnected small components. Therefore, to make the core graph interpretable, any components smaller than five nodes were removed.

We calculated the overall and diagnosis group specific network statistics using the following estimates: First, we report the total number of patients, number of professionals, median number of visits per professional, median number of visits per patient to each professional, and median number of visits per patient to each service type. Second, we reported the number of nodes (number of professionals) and the number of times that professionals shared at least five patients (number of edges). The other network indicators included, the mean degree centrality (average number of contacts between professionals), mean distance centrality (the average distance from a professional to all other professionals in the network), mean betweenness centrality (the extent to which a professional connects other two professionals), density (the number of connections with other professionals divided by the number of all possible connections within the network), transitivity (the probability that if professional A has shared patients with professionals B and C, then professionals B and C have also shared patients) and finally relative mean degree and relative mean betweenness of the occupational groups (average degree and betweenness centrality within the occupational group divided by the average degree or betweenness centrality in the other occupational groups). In order to allow for the comparison of each diagnosis-specific network to the all-patients network despite the number of patients differing, we randomly sampled the all-patients group down to the number of patients in each diagnosis group. We then created 100 iterations of each diagnosis group -sized network and used this distribution of values to test the null hypothesis that the group statistics were equal to the average network statistics of the sampled all-patients networks using one-sample Wilcoxon signed rank tests and nonparametric median-tests (Higgins, 2005).

We further analyzed the associations of the network structure and the nodal attributes (municipality, belonging to a certain occupational group) with patient sharing using Exponential Random Graph Models (ERGM; Frank & Strauss, 1986; Lusher et al., 2013). ERGMs predict the probability that a pair of nodes in a network will have a tie between them (in this case professionals sharing patients) given a set of persons and their attributes. In all ERGMs, we used the term edges that indicates the number of ties in the network to control for density, added the term to control for the main effects of the municipality, and another to capture the effects of working in the same municipality (uniform homophily). To gauge associations between occupation and patient sharing we included the main effect term of occupation (physicians, nurses, and others) and the term to capture the effects of belonging to the same occupational group (the occupation-based homophily) and the terms for testing the differential homophily effects of the occupational group. The Bayesian Information Criterion (BIC) was used to compare how well the hierarchical models fit to the data. The lower the BIC, the better the model fit.

The statistical analyses were made using R version 4.3.1 (R Core Team, 2024). We used the packages ‘tidyverse’ for data manipulation (Wickham et al., 2019), ‘igraph’ and ‘sna’ for network measures (Butts, 2023; Csardi& Nepusz, 2006), ‘ggraph’ for network visualizations (Pedersen, 2022), and ‘statnet’ (ergm) for exponential random graph modeling (Hunter et al., 2008).

## Results

The range of visits per patient was between 2 and 227 visits per year (median = 14 visits/year). The service types of visits varied from outpatient healthcare, such as long-term illness follow-up visits (62,892 visits), mental health and substance abuse related visits (44,792 visits), routine visits such as getting a vaccination (14,525), and other visits (25,221). Visits for professionals varied from 91,227 visits for nurses, 44,392 visits for physicians, and 11,811 visits for other professionals (Table 1).

**Table 1.** Network characteristics in all patients and each patient group.

Characteristics	All patients	Substance use disorder	*p*-value for difference	Psychotic disorders	*p*-value for difference	Depressive disorders	*p*-value for difference
Number of patients	8,217	870		513		2,187	
Number of visits /patients, median (IQR)	14 (8, 23)	20 (12, 32)	<.001	21 (13, 31)	<.001	17 (11, 25)	<.001
Number of professionals, *N* (%)							
Physicians	407 (26)	325 (32)		307 (34)		362 (28)	
Nurses	969 (62)	576 (58)		537 (58)		797 (61)	
Others	187 (12)	101 (10)		76 (8)		140 (11)	<.001
Number of visits in different professional							
Physicians	44,392 (30)	15,943 (27)		4,475 (34)		14,202 (32)	
Nurses	91,227 (62)	6,028 (71)		7,747 (59)		26,533 (60)	
Others	11,811 (8)	612 (2)		848 (7)		3,506 (8)	<.001
Number of visits/patients in each professional group, median (IQR)							
Physicians	5 (2, 8)	5 (3, 10)	<.001	7 (3, 13)	<.001	5 (3, 9)	<.001
Nurses	8 (5, 14)	13.5 (8, 22)	<.001	11 (7, 18)	<.001	10 (6, 15)	<.001
Others	2 (1, 6)	2 (1, 4)	.014	3 (1, 6)	.039	2 (1, 6)	.815
Number of visits each service type, *N* (%)							
GP/Primary	62,892 (43)	8,293 (37)		6,420 (49)		19,302 (44)	
Substance use psychiatric	44,792 (30)	11,285 (50)		4,598 (35)		14,157 (32)	
Routine (vaccinations etc.,)	25,221 (9)	1,525 (7)		1,011 (8)		6,821 (9)	
Other	14,525 (17)	1,480 (6)		1,041 (8)		3,961 (15)	<.001

**Figure 1.**
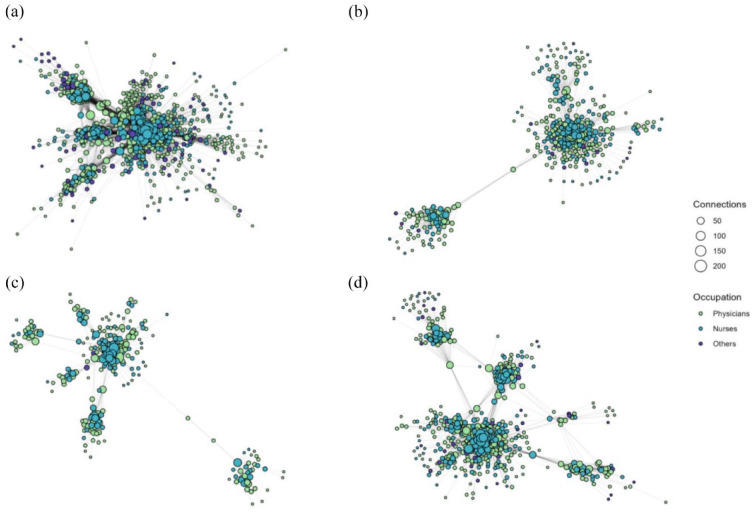
Patient sharing networks among health care professionals working in seven municipalities based on different patient groups: (a) all-patients network, (b) substance use network, (c) psychosis network, and (d) depressive network.

The networks illustrated in Figure 1 suggests that patient sharing was more prevalent within the seven municipalities than between them in the diagnosis-specific networks compared to the all-patients network. There also seemed to be some clustering within occupational groups with nurses usually most central and other groups in the peripheral. The network characteristics within patient diagnosis groups are shown in Table 1. The network sizes varied according to patient group sizes, but there were also clear differences in number of visits per patients and visits per patients to different professionals. The median number of visits per patient was larger in each diagnosis-specific groups compared to the all-patients group. Those with psychotic disorders, had visited health care professionals more frequently (median = 21 visits, IQR = 13–31) than those in the all-patients group. This was evident also when the visits were divided into visits to different professionals. Specifically, those with psychotic disorders had more visits with physicians (median = 7, IQR3 = –13 and nurses (median = 11, IQR = 7–18) than those in the all-patients group (median = 5, IQR = 2–8 and median = 8, IQR = 5–14, respectively).

After controlling for the total number of patients in the different networks, the number of professionals (nodes) and the number of edges connecting those professionals was greater in the diagnosis-specific networks compared to the corresponding sampled all-patients networks (Table 2). That also meant a higher average degree (more connections to other professionals) and more dense networks in diagnosis -specific groups. In the psychosis network the mean degree was 8.90, in the substance use network 14.37, and in the depressive network 23.07, when the corresponding figures in the sampled networks were 3.94, 6.89, and 18.24 (all *p* < .001). Mean betweenness was higher in substance use and psychosis networks, but lower in the depressive network compared to the corresponding sampled networks (Table 2).

**Table 2.** Network statistics by each patient group vs. all patients sampled mean (100 random samples with matching sizes to corresponding patient group).

Statistic	Substance use disorder			Psychotic disorders			Depressive disorders		
	All patient sampled mean	Patient group	*p* Value	All patient sampled mean	Patient group	*p* Value	All patient sampled mean	Patient group	*p* Value
Number of nodes	285.87	396	<.01	142.84	265	<.01	579.53	592	<.01
Number of edges	987.7	2,845	<.01	282.7	1,179	<.01	5,285.9	6,830	<.01
Mean degrees	6.89	14.37	<.01	3.94	8.9	<.01	18.24	23.07	<.01
Density	0.02	0.04	<.01	0.03	0.03	<.01	0.03	0.04	<.01
Mean distance	22.61	17.86	<.01	19.34	21.77	<.01	20.14	19.06	<.01
Transitivity	0.35	0.26	<.01	0.34	0.37	<.01	0.43	0.45	<.01
Mean betweenness	277.47	348.43	<.01	64.92	343.52	<.01	689.82	619.22	<.01
Relative betweenness									
Physicians	1.07	0.25	<.01	4.47	0.6	<.01	0.51	0.64	<.01
Nurses	1.54	4.73	<.01	0.6	1.74	<.01	2.83	2.06	<.01
Others	–	–	0.371	–	–		–	–	<.01
Relative degree									
Physicians	1.44	0.79	<.01	1.48	1.66	<.01	1.17	1.36	<.01
Nurses	0.78	1.47	<.01	0.76	0.63	<.01	1.07	0.96	<.01
Others	0.01	0.00	<.01	–	0.14	<.01	0.04	0.04	.157

Transitivity was lower in the substance use network but higher in the psychosis and depressive networks than in the sampled networks. Relative betweenness centrality differed across patient groups. Relative betweenness was higher in nurses and lower in physicians in the substance use and psychosis networks than in the all-patients network. In the depressive network the relative betweenness was higher in physicians and lower in nurses than in the all-patients networks (Table 2). In the all-patients networks, physicians had a higher tendency of within-occupation connections than other occupations (they had the highest relative degree). The relative degree in physicians was lower in the substance use network but higher in other diagnosis specific networks compared to the sampled all-patients networks (Table 2).

The degree to which each variable explains patient sharing in different patient group networks are reported in Table 3. Goodness of fit plots can be found in the Supplemental Materials (SFigure and SFigure 2). There were uniform associations of municipality with patient sharing in all networks, meaning that professionals working mainly in the same municipality tend to share patients with each other (Supplemental Tables 1 and 2). Nurses tended to share patients with fewer professionals than physicians in all networks except the substance use network in the second model. This is in line with the relative degree measures showing that nurses shared more patients in the substance use network but less in others. In the depressive disorders network having the same occupation was associated with a lower likelihood of patient sharing between two professionals. Conversely, in the all-patients and psychotic disorder networks, professionals shared patients more likely with those who had the same occupation as them. The differential homophily estimates suggested that physicians were more likely to share patients with other physicians in the substance use network but were less likely to share patients with other physicians in the depressive network. Nurses were more likely to share patients with other nurses in all patient network and less likely to share patients with other nurses in substance use disorder networks.

**Table 3.** Results of the ERGMs depicting the likelihood of a tie given professionals’ occupation group.

Occupational group	All patients	Substance use disorders	Psychotic disorders	Depressive disorders
	Log odds [95% CI]	Log odds [95% CI]	Log odds [95% CI]	Log odds [95% CI]
	Step1			
Physicians	Ref.	Ref.	Ref.	Ref.
Nurses	−0.37 [−0.40, −0.35]	−0.10 [−0.16, −0.05]	−0.53 [−0.62, −0.44]	−0.38 [−0.42, −0.35]
Other occupations	−1.13 [−1.18, −1.09]	−1.61 [−1.90, −1.33]	0.99 [ 0.72, 1.27]	−1.15 [−1.25, −1.04]
Uniform homophily: occupation	0.04 [0.01, 0.07]	0.07 [−0.01, 0.16]	0.33 [0.20, 0.46]	−0.07 [−0.12, −0.02]
AIC	162,822.59	19,719.86	7,441.91	44,943.56
BIC	162,944.94	19,821.80	7,535.00	45,054.36
	Step 2			
Physicians	Ref.	Ref.	Ref.	Ref.
Nurses	−0.46 [−0.54, −0.37]	0.71 [0.06, 1.36]	−0.40 [−0.92, 0.11]	−0.64 [−0.85, −0.44]
Other occupations	−1.20 [−1.26, −1.14]	−1.34 [−1.67, −1.02]	1.08 [ 0.69, 1.47]	−1.30 [−1.45, −1.15]
Differential homophily				
Physicians	−0.06 [−0.16, 0.03]	0.90 [0.24, 1.56]	0.47 [−0.07, 1.00]	−0.34 [−0.56, −0.13]
Nurses	0.12 [0.02, 0.21]	−0.75 [−1.40, −0.09]	0.20 [−0.34, 0.74]	0.20 [−0.02, 0.41]
Other occupations	0.51 [0.28, 0.73]	−	−0.29 [−2.75, 2.16]	0.48 [−0.21, 1.18]
AIC	162,806.59	19,714.58	7,445.41	44,938.79
BIC	162,951.19	19,825.77	7,555.43	45,069.73

*Note*. Log odds also adjusted for municipality (main effect and homophily).

In addition to traditional fit measures such as the BIC, we also assessed whether the network characteristics we assumed would drive tie formation reflect reality, that is, whether networks simulated based on the ERGMs resemble the observed data. We compared the observed data to 100 simulated networks derived from the fitted ERGMs (Model 1 in SFigure and Model 2 in SFigure 2). The three rows represent the statistics compared (Degree, edge-wise shared partners, and minimum geodesic distance), the four rows represent the different patient populations (all, substance use, psychosis, and depressive). Goodness-of-fit did not greatly differ between the two models. There were slight differences in fit across the patient groups. While the mean of the simulated network statistics seemed to occasionally deviate from the observed data, the networks seemed to follow similar overarching patterns especially considering the simplicity of the models where no dyad-dependent terms were included due to computation being infeasible in networks this large.

## Discussion

In the present study, we examined differences in patient-sharing between health care professionals taking care of mental and substance use disorder patients in the Finnish primary care during 1 year. We also examined the determinants of patient-sharing relationships between different professionals aiming to find out whether professionals’ attributes might explain patterns in professional co-operation in various patient groups. We found some differences in the patient characteristics and many of the network measures depending on the patient group. Patients in the diagnosis-specific networks had more visits and they visited a smaller group of professionals compared to all patients. Thus, the patient sharing networks were denser in substance use, psychotic, and depressive groups than in the all-patients network and, in the psychosis and depressive networks their patient sharing was also highly clustered, indicated by higher transitivity than in the all-patients network.

Networks with high connectivity have also been suggested to have high levels of co-operation and information sharing (Coleman et al., 1966; Creswick & Westbrook, 2010; Wensing et al., 2010). Our results could mean that in the diagnosis-specific networks and especially in the depressive network, co-operation, and information sharing would be higher than in the all-patients networks. Patient groups also seemed to differ in the relative centrality (betweenness) and number of connections (patients shared). Although the relative degree was high in physicians compared to other professionals, nurses were the most central occupation, and this was most evident in the substance use and depressive networks. These results support the idea of the Finnish primary health care system as a nurse-centered system. This may also partly reflect the central role of nurses in substance use replacement therapy and their relatively central role in providing psychotherapy and psychological counseling in the Finnish system (National Mental Health Strategy and Programme for Suicide Prevention 2020–2030). Recent results from the U.S. also seem to indicate that psychotherapy is no longer the most central task of the psychiatrists, since psychotherapy provided by psychiatrists has been dropped from 44.4% in the year 1996 to 21.6% in 2015 to 2016 (Tadmon & Olfson, 2022).

Our results suggest, firstly, that in the all-patients network, sharing patients was more likely when professionals belonged to the same occupation group. These results are in line with the homophily principle (the tendency of people to have contacts with similar others), which has been reported in many health care studies (Mascia et al., 2015). Homophilous relationships may be useful for the professionals when they enhance, for example, active information or advice sharing among peers. It has, however, been suggested that homophilous relationships (compared to heterophilous relationships between different professionals) do not encourage multidisciplinary teamwork and are undesirable from the perspective of health care organizations (Mascia et al., 2015).

In the depressive network uniform homophily was negative, suggesting that sharing patients with other professionals was more likely when they belonged to the different occupational groups. Thus, the depressive network seemed to reflect the multi-professional co-operation more evidently than other networks. This result is promising if it indicates that taking care of patients with mental health struggles is integrated within primary care services as is desired (WHO, 2021). The optimal patterns of multi-professional co-operation have been found to be dependent on the goal of the health care system (Lorant et al., 2017). If the main goal is to ensure continuity of care the networks are often constructed to be homophilous, whereas when the aim is to ensure social integration, the networks are heterophilous (Lorant et al., 2017). The optimal patient sharing network structure and architecture needs to be studied further in the future using a larger number of geographical and administrative areas and their patient sharing networks combined with longitudinal data on patients’ health status.

Unlike previous studies, we were able to examine the multidisciplinary networks in a real-world health care setting. As Hu et al. (2021) suggest, utilizing SNA among different professionals is probably the only way to focus to patient-centered interdisciplinary collaboration. Many previous patient-sharing studies using administrative data have also used claims data (DuGoff et al., 2018), which may lack some relevant information and reflect only the input of those professionals (physicians) who bill for their services (Yao et al., 2018). Thus, using a national register that includes all the different professionals working in primary care gives a more comprehensive and representative view to the patient-sharing relationships between professionals.

### Limitations

Our study also has limitations. First, we used data that included information only on a limited area in Finland and visits only from a 1-year time period. Studies in other areas in Finland and in other countries over longer period of time are needed to evaluate the reproducibility of our findings. Another challenge was how to reliably identify the patients who had visited primary care services for reasons related to mental health or substance abuse. The service type codes in the register generally reflect more on which organization the professionals operate under, rather than describe the nature of the visit (i.e. the needs of the patient). Following many previous studies using administrative data, we assumed that sharing patients generally indicates that the professionals also work together and share work-related information with each other. However, this interpretation cannot be confirmed by using the register data. Patients may visit several different professionals due to complex needs or referral policies within organizations. Some of the visits include also as additional information whether the visit included consultation some other professional or having meeting with some other professional. Of all the visits only 7.6% included such information.

The observed networks may also be the affected by some organization-induced changes, such as changes within service structures (due to COVID-19 or other reasons) or employee turnover; or some patient-induced factors, such as patient dissatisfaction with the received services, which might lead to seeking cervices from multiple sources for the same problem. Lastly, as suggested by previous studies, we defined that the professionals needed to share a certain number of patients (at least five) before they were really considered co-operating and that may have made our networks sparse, but that may be less of a problem than including random contacts.

We used data were from the Register of Primary Health Care visits (Avohilmo) covering all primary health care visits in Finland. This register was originally not collected for research purposes and thus have potential biases. However, the Finnish national register data are of high quality and collected directly and mandatorily from healthcare and education authorities. Resources are distributed based on visit data in Finland, so there are no reasons to over- or under report visits. One limitation is the risk of inaccuracies in diagnostic data. Diagnoses were supplied by clinicians and may, therefore, contain clinician-related bias. However, diagnostic practices have been shown to be reliable in Finnish psychiatric inpatient care (Sund, 2012) and as we analyzed the data on the level of diagnostic main classes, the risk of individual clinician-related bias is reduced. Although the quality of the Avohilmo register have shown to be very high (Sund, 2012), we checked data for any inconsistencies and impossible data values before the analyses.

Future studies should expand temporal scope to analyze the potential changes in the networks over time, when the future register follow-ups could be used. Similarly, integrating patient outcome measures to link network characteristics directly with patient care quality or outcomes would enhance the meaning of the network characteristics for policy and practice. Furthermore, including more municipalities or countries could improve the generalizability of the findings. Future studies could also collect qualitative data through interviews or focus groups with healthcare providers to enrich the understanding of why certain network patterns exist, and how they affect patient care.

## Conclusions

We found differences in the characteristics of patient-sharing networks depending on the patients’ main diagnosis. Diagnosis-specific patient-sharing networks were denser, more clustered, and characterized as potentially having more multi-occupational collaboration compared to networks including all patients with mental health or substance use disorders. In these networks nurses seemed to be the most central occupation. The biggest differences were between the depressive disorder patient network and the network including all patients with mental health or substance use disorders.

## Supplemental Material

sj-docx-1-isp-10.1177_00207640241270827 – Supplemental material for Variation in patient-sharing network characteristics of health care professionals treating different mental and substance use disorder patient sub-groups in primary care Supplemental material, sj-docx-1-isp-10.1177_00207640241270827 for Variation in patient-sharing network characteristics of health care professionals treating different mental and substance use disorder patient sub-groups in primary care by Marko Elovainio, Laura Hietapakka, Mai Gutvilig, Ripsa Niemi, Kaisla Komulainen, Laura Pulkki-Råback, Visa Väisänen, Timo Sinervo and Christian Hakulinen in International Journal of Social Psychiatry
