# Supplementary material for: Variation in patient-sharing network characteristics of health care professionals treating different mental and substance use disorder patient sub-groups in primary care
Source: Int J Soc Psychiatry. 2024 Aug 30;70(8):1442–52. doi: 10.1177/00207640241270827 (PMC11528958; doi:10.1177/00207640241270827)
Supplement: sj-docx-1-isp-10.1177_00207640241270827 – Supplemental material for Variation in patient-sharing network characteristics of health care professionals treating different mental and substance use disorder patient sub-groups in primary care [file sj-docx-1-isp-10.1177_00207640241270827.docx]

Supplement:

Marko Elovainio, Laura Hietapakka, Mai Gutvilig, Ripsa Niemi, Kaisla Komulainen, Laura Pulkki-Råback, Timo Sinervo, Christian Hakulinen.

Variation in patient-sharing network characteristics of health care professionals treating different mental and substance use disorder patient sub-groups in primary care

Stable 1. ERGM Analysis of occupational group and municipality (health center) main and homophility effects in four patient networks.

|  | All | Subst. | Psychotic | Depressive |
| --- | --- | --- | --- | --- |
| edges | -5.625 | -6.434 | -7.168 | -6.464 |
|  | [-5.683, -5.567] | [-6.638, -6.231] | [-7.486, -6.850] | [-6.601, -6.327] |
| Municipality 2 | 0.975 | 0.608 | 1.269 | 0.990 |
|  | [0.926, 1.023] | [0.399, 0.817] | [1.111, 1.426] | [0.920, 1.060] |
| Municipality 3 | 0.907 | 0.452 | 0.580 | 0.303 |
|  | [0.887, 0.927] | [0.399, 0.505] | [0.472, 0.687] | [0.243, 0.363] |
| Municipality 4 | 0.896 | 0.272 | 1.042 | 0.832 |
|  | [0.869, 0.924] | [0.180, 0.365] | [0.949, 1.136] | [0.790, 0.875] |
| Municipality 5 | 1.782 | 1.173 | 1.644 | 1.829 |
|  | [1.704, 1.860] | [0.976, 1.370] | [1.440, 1.848] | [1.684, 1.975] |
| Municipality 6 | 1.163 | 1.692 | 1.759 | 1.275 |
|  | [1.036, 1.290] | [1.374, 2.010] | [1.157, 2.360] | [0.999, 1.552] |
| Municipality 7 | 1.112 | 0.112 | 1.381 | 1.909 |
|  | [0.968, 1.255] | [-1.228, 1.451] | [0.751, 2.011] | [1.683, 2.136] |
| Uniform Homophily municipality | 3.861 | 4.058 | 4.739 | 4.489 |
|  | [3.809, 3.913] | [3.869, 4.248] | [4.442, 5.035] | [4.360, 4.619] |
| Main effect nurse | -0.375 | -0.105 | -0.533 | -0.383 |
|  | [-0.396, -0.354] | [-0.163, -0.047] | [-0.621, -0.444] | [-0.421, -0.345] |
| Main effect other occup. | -1.132 | -1.612 | 0.992 | -1.148 |
|  | [-1.177, -1.087] | [-1.895, -1.329] | [0.717, 1.268] | [-1.253, -1.043] |
| Uniform Homophily occupation | 0.039 | 0.075 | 0.328 | -0.068 |
|  | [0.009, 0.068] | [-0.006, 0.156] | [0.198, 0.458] | [-0.122, -0.015] |
| AIC | 162822.6 | 19719.9 | 7441.9 | 44943.6 |
| BIC | 162944.9 | 19821.8 | 7535.0 | 45054.4 |

STable 2. ERGM Analysis of occupational group and municipality (health center) main and homophily effects in four patient networks

|  | All | Subst. | Psychotic | Depressive |
| --- | --- | --- | --- | --- |
| edges | -5.536 | -7.251 | -7.303 | -6.202 |
|  | [-5.637, -5.434] | [-7.931, -6.570] | [-7.908, -6.698] | [-6.443, -5.961] |
| Municipality 2 | 0.975 | 0.608 | 1.270 | 0.990 |
|  | [0.926, 1.023] | [0.399, 0.817] | [1.113, 1.428] | [0.920, 1.060] |
| Municipality 3 | 0.907 | 0.452 | 0.581 | 0.303 |
|  | [0.887, 0.927] | [0.399, 0.505] | [0.473, 0.689] | [0.243, 0.363] |
| Municipality 4 | 0.896 | 0.272 | 1.044 | 0.832 |
|  | [0.869, 0.924] | [0.179, 0.364] | [0.950, 1.137] | [0.790, 0.875] |
| Municipality 5 | 1.782 | 1.173 | 1.645 | 1.830 |
|  | [1.704, 1.860] | [0.976, 1.370] | [1.441, 1.849] | [1.684, 1.975] |
| Municipality 6 | 1.163 | 1.692 | 1.760 | 1.275 |
|  | [1.036, 1.290] | [1.374, 2.010] | [1.159, 2.362] | [0.998, 1.551] |
| Municipality 7 | 1.111 | 0.109 | 1.382 | 1.909 |
|  | [0.968, 1.255] | [-1.230, 1.449] | [0.752, 2.012] | [1.682, 2.135] |
| Uniform Homophily municipality | 3.861 | 4.058 | 4.740 | 4.489 |
|  | [3.809, 3.914] | [3.869, 4.248] | [4.444, 5.037] | [4.360, 4.618] |
| Main effect nurse | -0.457 | 0.711 | -0.402 | -0.643 |
|  | [-0.543, -0.371] | [0.059, 1.363] | [-0.917, 0.114] | [-0.845, -0.442] |
| Main effect other occup. | -1.199 | -1.344 | 1.081 | -1.298 |
|  | [-1.258, -1.141] | [-1.667, -1.020] | [0.688, 1.474] | [-1.449, -1.147] |
| Differential Homophily: |  |  |  |  |
| Physicians | -0.065 | 0.902 | 0.465 | -0.344 |
|  | [-0.160, 0.031] | [0.241, 1.563] | [-0.074, 1.004] | [-0.557, -0.130] |
| Nurses | 0.116 | -0.745 | 0.196 | 0.196 |
|  | [0.025, 0.207] | [-1.402, -0.088] | [-0.344, 0.735] | [-0.015, 0.407] |
| Other occupations | 0.506 |  | -0.293 | 0.484 |
|  | [0.278, 0.734] |  | [-2.750, 2.165] | [-0.212, 1.181] |
| AIC | 162806.6 | 19714.6 | 7445.4 | 44938.8 |
| BIC | 162951.2 | 19825.8 | 7555.4 | 45069.7 |


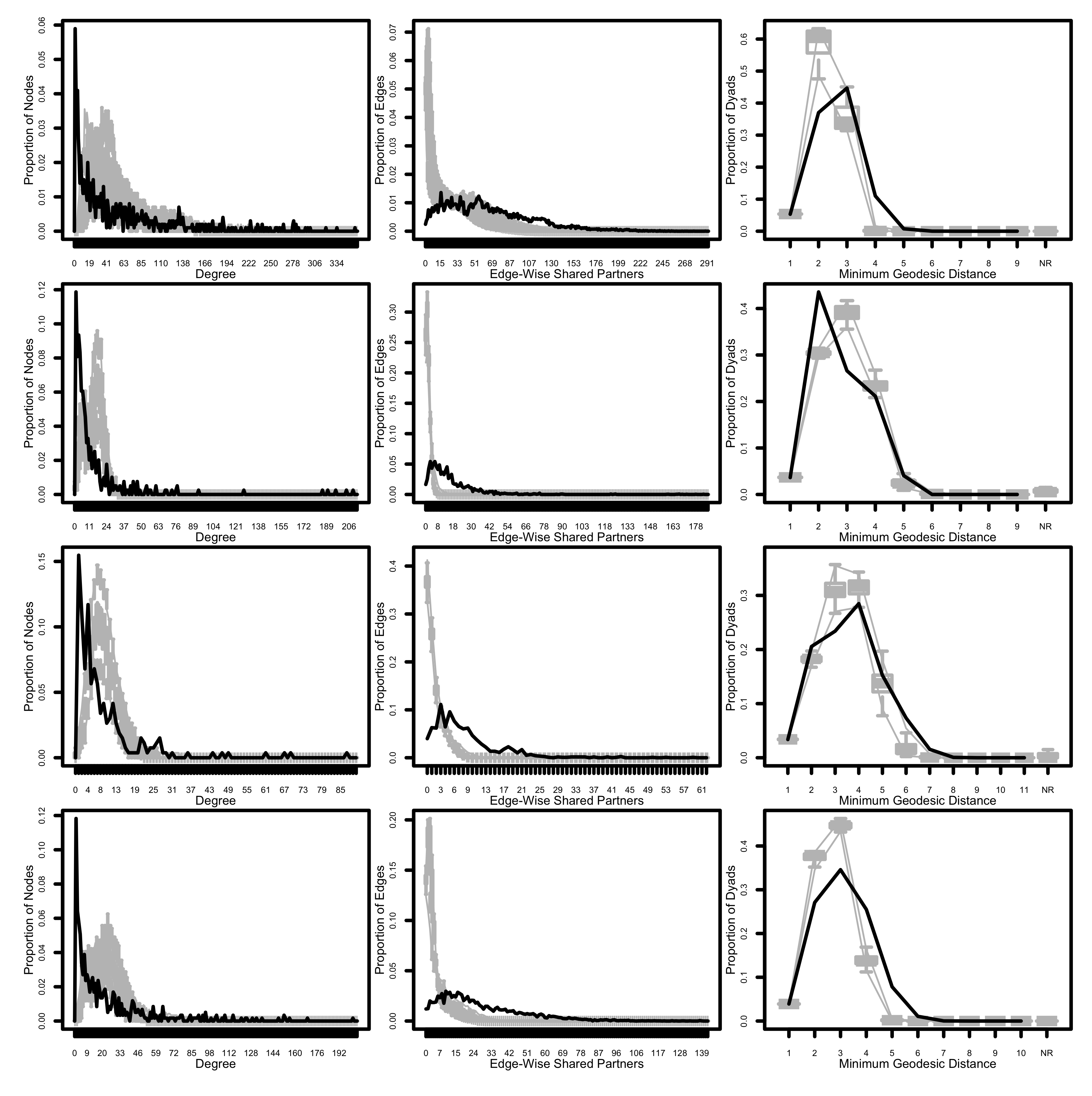


SFigure 1. Goodness of fits of the ERGM step 1


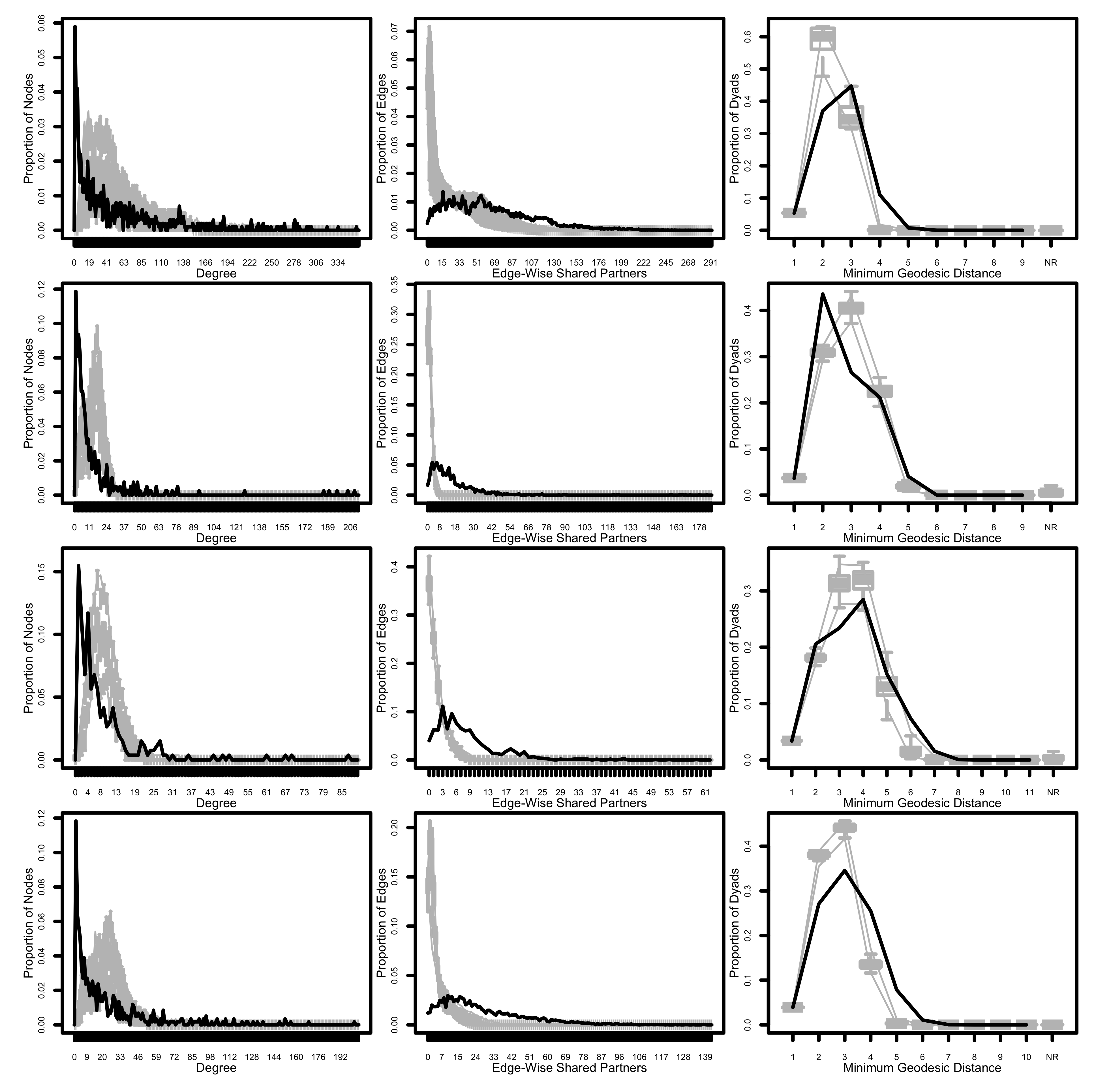


SFigure 2. Goodness of fits of the ERGM step 1
